# Validation of diagnoses of liver disorders in users of systemic azole antifungal medication in Sweden

**DOI:** 10.1186/s12876-023-03110-w

**Published:** 2024-01-05

**Authors:** Diego Hernan Giunta, Pär Karlsson, Muhammad Younus, Ina Anveden Berglind, Helle Kieler, Johan Reutfors

**Affiliations:** 1 Centre for Pharmacoepidemiology, Karolinska Institutet, Karolinska University Hospital T2:02, 171 76, Stockholm, Sweden; 2 Safety Surveillance Research, Worldwide Medical and Safety, Pfizer Inc, Collegeville, PA USA; 3 Center for Occupational and Environmental Medicine, Stockholm Region, Stockholm, Sweden

**Keywords:** Validation, Positive predictive value, Drug induced liver injury, Acute liver injury, Liver disorders, Toxic liver disease, Hepatic failure, Jaundice

## Abstract

**Background:**

Liver disorders are important adverse effects associated with antifungal drug treatment. However, the accuracy of Clinical International Classification of Diseases (ICD)-10 codes in identifying liver disorders for register based research is not well-established. This study aimed to determine the positive predictive value (PPV) of the ICD-10 codes for identifying patients with toxic liver disease, hepatic failure, and jaundice among patients with systemic antifungal treatment.

**Methods:**

Data from the Swedish Prescribed Drug Register and the National Patient Register were utilized to identify adult patients who received systemic azole antifungal drugs and had a recorded diagnosis of toxic liver disease (K71.0, K71.1, K71.2, K71.6, K71.8, K71.9), hepatic failure (K72.0, K72.9), or jaundice (R17) between 2005 and 2016. The medical records of all included patients were reviewed. Prespecified criteria were used to re-evaluate and confirm each diagnosis, serving as the gold standard to calculate PPVs with 95% confidence intervals (95% CI) for each diagnostic group.

**Results:**

Among the 115 included patients, 26 were diagnosed with toxic liver disease, 58 with hepatic failure, and 31 with jaundice. Toxic liver disease was confirmed in 14 out of 26 patients, yielding a PPV of 53.8% (95% CI 33.4–73.4%). Hepatic failure was confirmed in 26 out of 38 patients, resulting in a PPV of 62.1% (95% CI 48.4–74.5%). The highest PPV was found in jaundice, with 30 confirmed diagnoses out of 31, yielding a PPV of 96.8% (95% CI 83.3–99.9%).

**Conclusion:**

Among patients who received azole antifungal treatment and were subsequently diagnosed with a liver disorder, the PPV for the diagnosis of jaundice was high, while the PPVs for toxic liver disease and hepatic failure were lower.

## Background

Invasive fungal infections can lead to serious morbidity and mortality in immunocompromised and severely ill patients [1], with attributable mortality rates of up to 39% [2, 3]. Cancer patients with chemotherapy-induced neutropenia and transplant recipients receiving immunosuppressive therapy are particularly susceptible to invasive fungal infections, resulting in an increased use of systemic antifungal agents to prevent or treat these infections [4–7]. However, the use of systemic antifungal agents may affect the liver function, leading to hepatic functional abnormalities ranging from transient mild liver injury to severe hepatic reactions including hepatitis, cholestasis and fulminant hepatic failure, even among patients with no other identifiable risk factors [8]. Liver dysfunction and symptoms due to antifungal agents’ exposure is in general reversed when treatment is discontinued, but fatal events have been reported [6, 9].

Toxic liver disease, also known as drug-induced liver injury (DILI), is a hepatic injury associated with drug exposure that can vary in hepatotoxicity mechanism and clinical presentation (hepatocellular, cholestatic, mixed), as well as histologic findings (hepatitis, cholestasis, steatosis) [10, 11]. Liver function tests (serum alanine transaminase, serum aspartate transaminase, alkaline phosphatase or bilirubin) are used to identify toxic liver disease, which generally leads to drug discontinuation when other alternative causes of liver injury have been excluded [6]. Acute hepatic failure with a rapid onset of coagulopathy and hepatic encephalopathy can also be caused by drugs, including antifungals [12]. Coagulopathy can include diminished synthesis of procoagulant factors, impaired anticoagulant and fibrinolytic systems, as well as defective function and number of platelets [13]. Hepatic encephalopathy involves a broad spectrum of neuropsychiatric abnormalities ranging from subclinical alterations to coma [14]. Jaundice, a yellow pigmentation of the skin and sclera due to high bilirubin levels, can be the first or only sign of liver disease, also when associated with drug exposure, and is therefore a relevant outcome in this context [15].

Routinely collected clinical data such as discharge diagnoses recorded in secondary databases and registries is becoming an increasingly important source of real-world information for research and to monitor and detect potential safety signals [16, 17]. Previous validation studies for liver injury or hepatic failure have used combinations of laboratory test results and diagnostic codes according to the 10th International Statistical Classification of Diseases and Related Health Problems (ICD-10) [18–20]. However, since laboratory data may not always be available, it is essential to establish the validity of ICD-10 codes to identify cases of liver disorder. Comprehensive clinical data from medical records, can be used to assess the validity of diagnoses recorded in secondary databases.

Our objective was to estimate the positive predictive value (PPV) of ICD-10 codes to identify patients with toxic liver disease, hepatic failure, and jaundice among patients with azole systemic antifungal treatment by using data from medical records as the gold standard for validation.

## Methods

### Data sources

All medical diagnoses recorded at hospital discharges and at specialized outpatient clinics are included in the Swedish National Patient Register (NPR) [21]. Diagnoses are coded using ICD-10 codes since 1997 [22]. All prescribed dispensed medications for the entire Swedish population, excluding medications administered during hospitalisation, are registered in the Swedish Prescribed Drug Register (PDR), [23]. The drugs are classified using Anatomical Therapeutic Chemical (ATC) classification code, including the dispensing date and the amount in defined daily doses (DDD) [24]. We linked information from these two data sources using the Swedish 12-digit personal identity number [25]. Information on liver disorder diagnosis was obtained from medical records, using predefined criteria as described in the following sections.

To gain access to the medical records, we contacted 77 clinics where the patients had been treated according to the NPR, covering all regions in Sweden. The request included a description of the study and its ethical approval, with a detailed request for access to the medical records. The medical records included physician notes, laboratory results (including autoimmune and virologic results), diagnostic imaging results, and pathology reports.

### Study design and population

Using nationwide Swedish databases from July 2005 to September 2016, we included patients who had at least one filled prescription for any systemic antifungal drug (ATC-code J02A) recorded in the PDR, representing seven or more defined daily doses (DDD), and who had at least one ICD-10 code for toxic liver disease (K71.0, K71.1, K71.2, K71.6, K71.8, K71.9), hepatic failure (K72.0, K72.9), or jaundice (R17) as a primary or secondary diagnosis in the NPR, after the first filled prescription of the azole antifungal drug (Table 1**)** This liver disorders were chosen to represent different degrees of potentially drug related treatments. The time between filling a prescription of an antifungal drug and the liver disorder diagnosis had to be less than 6 months. Included antifungal drugs were imidazole derivates (J02AB02 ketoconazole) and triazoles (J02AC01 fluconazole, J02AC02 itraconazole, J02AC03 voriconazole, and J02AC04 posaconazole). We excluded patients with any primary or secondary diagnosis of toxic liver disease, hepatic failure, or jaundice in the NPR within 5 years prior to the first filling of a prescription of an antifungal drug. If a patient had two or more diagnoses that fell into more than one stratum at a single visit, such a patient was included in all relevant diagnostic strata.

**Table 1:** 10th International Statistical Classification of Diseases and Related Health Problems ICD-10 codes for liver disorders

ICD-9 code	ICD-9 diagnosis code description
**Toxic liver disease**	
K71.0	Toxic liver disease with cholestasis
K71.1	Toxic liver disease with hepatic necrosis
K71.2	Toxic liver disease with acute hepatitis
K71.6	Toxic liver disease with hepatitis, not elsewhere classified
K71.8	Toxic liver disease with other disorders of liver
K71.9	Toxic liver disease, unspecified
**Hepatic failure**	
K72.0	Acute and subacute hepatic failure
K72.9	Hepatic failure, unspecified
**Jaundice**	
R17	Unspecified jaundice

With the aim of reviewing at least 100 medical records for patients fulfilling the inclusion criteria, we used stratified random sampling without replacement stratified by each one of the 3 liver disorders, aiming to draw 40% of the total sample from the records for patients with toxic liver disease, 40% from records for patients with hepatic failure, and 20% from records for patients with jaundice to represent cases with the three diagnostic categories of interest. As individual clinics determined whether the requested medical records, could be made available for this study, an additional 50 records were drawn from the sample to make sure that at least 100 records could be reviewed. If a patient was recorded more than once in the NPR with a liver disorder diagnosis, one of the recorded visits was selected by a simple random sampling.

### Validation criteria for liver disorder diagnosis

The “gold standard” operational definition of each diagnosis was defined according to feasibility and available information according to the following predefined definitions in the research protocol:

#### Toxic liver disease

Since there is no specific diagnostic test confirming toxic liver disease, exclusion of other conditions that can cause liver injury was used to confirm the diagnosis. As a tool for this, we used the *Roussel-Uclaf Causality Assessment Method* (RUCAM) [26]. Although RUCAM is rarely used in daily clinical practice because it is cumbersome, it has been shown to have high specificity, reliability, and reproducibility [27–29]. The parameters included in the RUCAM include chemistry, immunology, virology, radiology, preexisting liver disease, sepsis, metastatic malignancy, alcohol, and pregnancy*.* However, as RUCAM is not fully applicable clinically, we also used a modified RUCAM by adding customized points, including: 1) histological liver biopsy findings typical for toxic drug effect as reflected by high scores and 2) the treating physician’s assessment of disease severity.

#### Hepatic failure

To define hepatic failure, we used three definitions including the presence of coagulopathy as an indicator of impairment of liver synthetic function from the laboratory results, the presence of ascites and/or encephalopathy as follows: 1) International definition of hepatic failure (coagulopathy with an International Normalized Ratio (INR) of > 1.5, liver encephalopathy within 8 weeks from diagnosis of liver disease, and a disease duration shorter than 26 weeks from diagnosis of liver disease) [12, 30]; 2) Liver synthetic dysfunction (coagulopathy with INR over the upper limit, bilirubin over the upper normal limit, and serum albumin under the lower limit) without ascites; and 3) Liver synthetic dysfunction with ascites.

#### Jaundice

Because jaundice is a sign with a laboratory confirmation, rather than a disease in itself, the ICD-10 code for jaundice is non-specific. We used two definitions for the gold standard for jaundice in the medical records: 1) only a laboratory result with a serum bilirubin over 80 μmol/L, or 2) serum bilirubin over 35 and less or equal to 80 μmol/L plus a description of clinical findings of jaundice in skin or sclera at clinical inspection.

### Description of the validation process

The patients were order randomly, and trained reviewers scrutinized the medical records to systematically collect relevant information about the three diagnoses of interest recorded from hospitalizations and outpatient visits, using structured forms. There were three reviewers, CB being a registered nurse, JR a medical doctor, and ME a medical doctor and specialist in gastroenterology and hepatology. Laboratory tests, radiology examinations, histology reports, and immunology examinations were also reviewed and variables relevant to the different disorders were manually abstracted and transferred into the computerized abstraction form prepared for each diagnostic group in a Microsoft Access Database. In the same software and in SAS 9.4, computerized algorithms based on internationally used clinical criteria were implemented to categorize the diagnosis of interest as ‘confirm’ or ‘did not confirm’ based on strict criteria for each diagnosis. In case of inconsistency between the diagnostic output of the computerized algorithm and the gastroenterology specialist, the discrepancy was reconciled by consensus among the three reviewers. This occurred for 12 out of 26 patients with toxic liver disease, for 20 out of 58 patients with hepatic failure, and for 2 out of 31 patients with jaundice. Any type of malignancy (ICD-10 Neoplasms C00-D48) diagnosed in a 10 years window prior to the date of the liver disorder diagnosis was explored as proxies for background disease severity for each patient.

### Statistical analyses

PPV was defined as the probability that a patient diagnosed with hepatic failure, toxic liver disease or jaundice based on the ICD-10 classification would be classified within the same diagnostic group using medical record review as the gold standard. Thus, PPV was calculated by dividing the number of patients who met diagnostic criteria by medical record review (true positives), by the number of patients with a recorded ICD-10 code in the NPR. Since K71.1 is the ICD-10 code for toxic liver disease with hepatic necrosis, a severe toxic liver disease resulting in hepatic failure, for toxic liver disease we conducted the analyses with and without the inclusion of K71.1. The PPVs for the individual codes are presented with 95% confidence intervals (95% CI) calculated using the Clopper-Pearson binomial exact method [31].

## Results

**Fig. 1:**
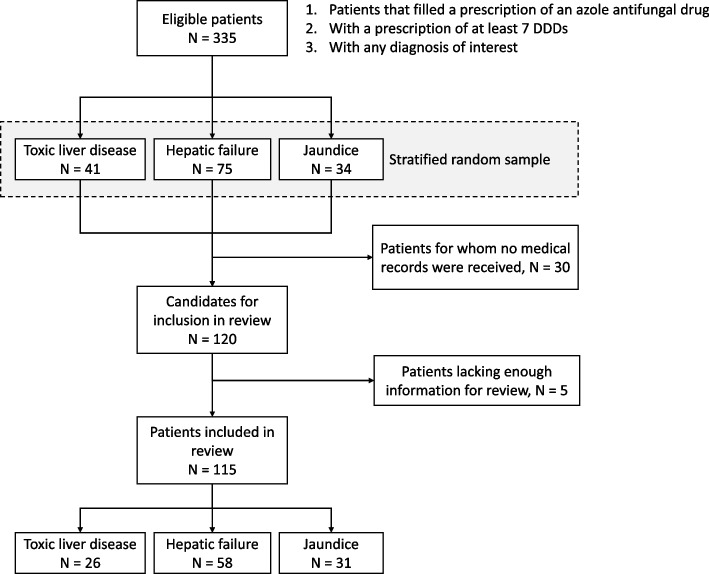
Flow chart for identification of the study population

A total of 557,809 patients who had filled at least one prescription of any antifungal drug were identified. Among them, 117,591 patients (21%) had at least one filled prescription corresponding to seven or more DDDs. Out of these patients, 1523 (1%) were also found in the NPR at least once with a primary or secondary ICD-10 diagnosis of toxic liver disease, hepatic failure, or jaundice. There were 335 eligible patients identified with any of these diagnosis within 6 months after their first filled prescription of an antifungal drug, and we sampled 150 eligible patients who fulfilled our inclusion criteria (Fig. 1). After exclusion of incomplete medical records, a final total of 115 patients were included in the study, consisting of 26 with toxic liver disease, 58 with hepatic failure, and 31 with jaundice.

At the time of liver disorder diagnosis, 97 (84.3%) patients were using fluconazole, 7 (6.1%) ketoconazole, 6 (5.2%) voriconazole, 4 (3.5%) itraconazole, and 1 (0.9%) posaconazole. Table 2 presents the demographic characteristics and medical history of the 115 patients. Malignancy within 10 years prior to the liver disorder was common: 16 (62%), 39 (67%) and 26 (84%) in patients with toxic liver disease, hepatic failure or jaundice, respectively.

**Table 2:** Characteristics of the study population

		Toxic liver disease (*N* = 26)	Hepatic failure (*N* = 58)	Jaundice (*N* = 31)
		*n* (%)	*n* (%)	*n* (%)
Sex	Male	11 (42%)	36 (62%)	15 (48%)
	Female	15 (58%)	22 (38%)	16 (52%)
Age (years)	0–17	4 (15%)	3 (5%)	0
	18–39	5 (19%)	4 (7%)	1 (3%)
	40–59	12 (46%)	14 (24%)	11 (35%)
	60–69	5 (19%)	24 (41%)	10 (32%)
	> 70	0	13 (22%)	9 (29%)
Calendar year of diagnosis	2005–2007	6 (23%)	1 (2%)	4 (13%)
	2008–2010	5 (19%)	18 (31%)	8 (26%)
	2011–2012	10 (38%)	17 (29%)	9 (29%)
	2013–2014	5 (19%)	22 (38%)	10 (32%)
Any malignancy 10 years prior to liver disorder diagnosis		16 (62%)	39 (67%)	26 (84%)
Antifungal azole drug prior to liver disorder diagnosis	Ketoconazole	2 (8%)	4 (7%)	1 (3%)
	Fluconazole	23 (88%)	45 (78%)	29 (94%)
	Itraconazole	1 (4%)	3 (5%)	0
	Voriconazole	0	5 (9%)	1 (3%)
	Posaconazole	0	1 (2%)	0

Among the 26 patients with ICD-10 codes for toxic liver disease, the diagnosis was confirmed for 14 patients*.* The PPV for a confirmed diagnosis of toxic liver disease was 53.8% (95% CI 33.4–73.4%), as presented in Table 3. Of the confirmed cases, 7 patients had undergone liver biopsy, which supported the diagnosis of toxic liver disease in 6 cases.

**Table 3:** PPVs for toxic liver disease, hepatic failure without and with toxic liver disease with hepatic necrosis, and jaundice

	Evaluable patients *N*	Confirmed diagnosis (true positive) *N*	Non-confirmed diagnosis (false positives) *N*	PPV (95% CI)
**Toxic liver disease**	26	14	12	53.8% (33.4–73.4%)
**Hepatic failure**				
Any hepatic failure	58	36	22	62.1% (48.4–74.5%)
Liver synthetic dysfunction	58	31	27	53.4% (39.9–66.7%)
Liver synthetic dysfunction with ascites	58	18	40	31.0% (19.5–44.5%)
International definition of hepatic failure	58	21	37	36.2% (24.0–49.9%)
**Hepatic failure including toxic liver disease with hepatic necrosis**^**a**^				
Any hepatic failure	61	37	24	60.7% (47.3–72.9%)
Liver synthetic dysfunction	61	32	29	52.5% (39.3–65.4%)
Liver synthetic dysfunction with ascites	61	19	42	31.1% (19.9–44.3%)
International definition of hepatic failure	61	21	40	34.4% (22.7–47.7%)
**Jaundice**	31	30	1	96.8% (83.3–99.9%)

a. Toxic liver disease with hepatic necrosis - ICD-10 code K71.1

Out of the 58 patients with ICD-10 codes for hepatic failure, the diagnosis was confirmed in 36 patients*.* The PPV for a confirmed diagnosis of any hepatic failure was 62.1% (95% CI 48.4–74.5%). Regarding the subgroups of hepatic failure based on the different operational definitions used in this study, the PPV was highest for liver synthetic dysfunction, followed by the international definition of hepatic failure and liver synthetic dysfunction with ascites. However, the confidence intervals for the different definitions overlapped.

Of the 3 patients with the diagnosis K71.1 (toxic liver disease with hepatic necrosis), only one met the diagnostic criteria for hepatic failure. In the sub analysis including K71.1 for any hepatic failure, the PPV for a confirmed diagnosis was 60.7% (47.3–72.9%). The PPVs for the diagnostic subgroups were similar to for the analyses where K71.1 was not included. All PPV for hepatic failure are presented in Table 3.

Out of the 31 patients with an ICD-10 code for jaundice, the diagnosis was confirmed in 30 patients*.* The PPV for a confirmed diagnosis of jaundice was 96.8% (95% CI 83.3–99.9%), as shown in Table 3.

## Discussion

This study aimed to evaluate the validity of ICD-10 diagnoses for toxic liver disease, hepatic failure, and jaundice among patients treated with antifungal drugs by reviewing their medical records. We observed varying PPVs across the diagnoses, with a high PPV of 96.8% for jaundice, but lower PPVs of 62.1% for hepatic failure and 53.8% for toxic liver disease.

Assessing the validity of diagnoses by comparing them to the gold standard of medical record diagnosis is crucial in real-world studies that utilize secondary databases to understand the risk of misclassification [32]. Few previous studies have reported the validity of ICD-10 diagnoses of toxic liver disease in population-based secondary databases [18, 33, 34], and none have specifically examined patients receiving systemic antifungal treatment.

The PPVs for toxic liver disease in the present study are within the range as reported in previous studies estimating the PPV for acute liver injury. However, the codes, populations, and methods used in these studies are not homogenous, making direct comparisons of results challenging [18, 34, 35]. Forns et al. conducted a validation study using ICD-10 and ICD-9 codes for acute liver injury in adult incident antidepressant users from Denmark, Spain, and Germany, excluding patients with preexisting liver disease or other risk factors [33, 34]. They employed two sets of codes, with specific codes similar to the ICD codes included in the present study for acute liver injury and hepatic failure together, while nonspecific codes included jaundice, hepatomegaly, hepatic enzyme alteration, and liver transplant. The PPVs for specific codes using hospital and outpatient diagnosis ranged between 34.8 to 83.3%. A related study from Germany using the same specific and nonspecific ICD-10-GM codes in hospital-discharged patients reported PPVs for specific codes of 48.7% for all patients and 63.0% after excluding patients with known cancer, chronic liver, biliary and pancreatic disease, heart failure, and alcohol-related disorders [35]. Both studies demonstrated lower PPVs for nonspecific codes and outpatient diagnosis, with overlapping CIs compared to our study. Another validation study used electronic health records in Michigan to identify patients with idiosyncratic drug induced liver injury [18], usingK71 (toxic liver injury) along with ICD-10 codes for 15 specific drug poisonings and concomitant laboratory criteria. The estimated PPV was 66.5%, which is also similar to our findings. These results suggest that the use of less frequent conditions codes, such as drug-specific ICD codes, nonspecific codes, or the use of the laboratory results do not markedly improve PPV.

The PPV of 62.1% for hepatic failure in our study was higher than that reported in previous validation studies that combined ICD codes with laboratory results. One study that aimed to identify acute hepatic failure using a combination of hospital ICD-9 diagnosis and laboratory results (presence of coagulopathy and hyperbilirubinemia) reported PPVs between 5 and 15% [19]. Similarly, another study identified hepatic failure using a combination of inpatient or outpatient ICD-10 K72 codes and at least one of 15 T-codes for drug toxicity/poisoning, along with concomitant laboratory criteria for clinically significant liver injury, to detect idiosyncratic toxic liver disease with hepatic failure. They also observed low PPV between 10.7 and 15% [20]. This last validation study used a similar code definition as the one we used to identify hepatic failure, but since both studies used more strict definitions with laboratory results, comparing PPVs may not be appropriate.

In the above-mentioned study by Forns et al., jaundice was considered a nonspecific diagnosis for identifying acute liver injury, resulting in PPVs ranging from 35.1% in Spain to 94.9% in Denmark [34]. Our results are similar to the estimated PPV for Denmark possibly due to the similarities between the two countries in terms of health care systems and national registries. The finding that the non-specific diagnosis of jaundice had the highest PPV for detecting jaundice was expected, since it merely describes a symptom or sign, making it an easy diagnosis, and the information supporting the diagnosis was readily available in the medical records. However, it should be noted that jaundice per se can be caused by hepatic, prehepatic, and post-hepatic causes, and is not always a sign of liver disease [36].

Several limitations of this study should be considered when interpreting the findings. Since only ambulatory dispensed drugs are available in the PDR, only azole antifungal drug treatments were included in this study. The lack of blinding of the reviewers, since all medical records corresponded to patients with one of the ICD-10 codes under study, may have resulted in an overestimation of the PPVs [37]. Additionally, a relatively small sample size for each specific liver diagnosis limited the precision of the PPV estimates, leading to broad confidence intervals. Furthermore, the three gold standard definitions used to classify the liver diagnoses are not perfect, as the attending clinician may have had additional information not documented in the medical records. Some cases of transient, self-limited, and low clinical impact hepatotoxicity may not have been diagnosed. As for the assessment of toxic liver disease, the selected assessment tool, RUCAM, is designed to evaluate general toxic effects of a drug on the liver, indicating the likelihood that liver injury is caused by a specific medication [38, 39]. We chose RUCAM because it is widely used for this purpose. However, the diagnosis of toxic liver disease itself relies on expert assessment. The patients diagnosed with toxic liver disease had a significant number of missing laboratory results, meaning that some of the information used by attending clinicians was not available in the medical record review. These factors likely decreased the proportion of patients for whom the diagnosis could be confirmed, resulting in lower estimated PPV.

The criteria for hepatic failure may vary between countries, and to highlight such differences, we employed three different definitions. According to the international criteria for hepatic failure, liver function tests (LFTs) must remain pathological for at least 26 weeks. To determine if the patients in our sample met these criteria, we requested medical records up to 6 months after the diagnosis. However, medical records were only available from inpatient care or hospital ambulatory care, and LFTs from primary care sources were unavailable. Therefore, there was no available information on LFTs for the full time period of 26 weeks after diagnosis in the majority of patients. Consequently, most patients could not be confirmed to have met the criteria for the international definition of hepatic failure. Another factor that likely decreased the proportion of patients with confirmed hepatic failure is that diagnosing encephalopathy is not straightforward and may be under-recorded.

Among patients with serious underlying medical conditions, abnormal LFTs and liver disease may be attributed to their medical conditions or concomitant treatments. In the present study, all patients were prescribed antifungal drugs and were severely ill. In fact, 70% of patients had been diagnosed with malignancies within 10 years prior to the reported liver disease diagnosis, and 69% died within 1 year after the diagnosis of liver disease. It is possible that organ-specific ICD-10 codes such as hepatic failure were used non-specifically in this severally ill population, and therefore the necessary criterion to support a formal hepatic failure diagnosis in our validation may not have been recorded, or even measured.

## Conclusion

Among patients treated with systemic azole antifungal drugs and subsequently diagnosed with liver disorders, the PPVs for toxic liver disease, hepatic failure, and jaundice ranged from 53.8 to 96.8%. The lower PPVs observed for toxic liver injury and hepatic failure may reflect that the multifaceted nature of these diagnoses compared to the diagnosis of jaundice. Our study provides insights into the validity of liver injury diagnoses when utilizing the Swedish national registries and secondary population-based databases. These findings are likely applicable to patients treated with antifungals and diagnosed with liver disorder in Sweden or similar populations.

## Data Availability

The datasets generated and/or analysed during the current study are not publicly available due [REASON WHY DATA ARE NOT PUBLIC] but are available from the corresponding author on reasonable request.

## Abbreviations

ATC: Anatomical therapeutic chemical
CI: Confidence intervals
DDD: Defined daily dose
DILI: Drug-induced liver injury
ICD-10: 10th International Statistical Classification of Diseases and Related Health Problems
LFT: Liver function tests
NPR: Swedish national patient register
PASS: Post-authorization safety study
PDR: Swedish prescribed drug register
PPV: Positive predictive value
RUCAM: Roussel-uclaf causality assessment method
